# Kikuchi-Fujimoto Disease: The Unexpected Diagnosis of a Cervical Adenopathy

**DOI:** 10.7759/cureus.42091

**Published:** 2023-07-18

**Authors:** Liliana Santa Cruz, Ana Sofia Martins, Ana Salomé Guedes, Mariana Mendonça, Liliane Carvalho

**Affiliations:** 1 General and Family Medicine, USF (Unidade de Saúde Familiar) Coimbra Sul, Coimbra, PRT; 2 General and Family Medicine, USF (Unidade de Saúde Familiar) Mondego, Coimbra, PRT

**Keywords:** primary healthcare, necrotizing, lymphadenitis, adenopathy, kikuchi-fujimoto, case reports

## Abstract

Many cases of adenopathies, whose differential diagnosis includes a wide spectrum of pathologies (including some malignant conditions like lymphoproliferative diseases, e.g., lymphomas), resort to primary healthcare. Kikuchi-Fujimoto disease is a rare, benign, self-limiting entity characterized by adenopathies, mainly in the cervical region, which may be associated with constitutional symptoms. This specific pathology is very rare in primary care and is often overlooked. That is why it is essential to promote medical literacy and provide support in managing these cases, which we want to emphasize through this case presentation. This case report presents a 24-year-old female patient who sought a consultation at the Family Health Unit due to a painful swelling in the right cervical region that lasted two weeks. She denied a history of recent infections or constitutional symptoms. A painful and hard right submaxillary mass, measuring 2 cm in diameter, was identified upon palpation. An analytical study and ultrasound of the soft tissues of the cervical region were initially required. Analytically, there were no relevant changes; however, the ultrasound revealed “hypoechoic ganglion formations in the right laterocervical chains, from the retroauricular region to the lower region of the neck, the largest measuring 19x7mm”. The patient was reassessed one month later, due to an increase in the number of adenopathies, and a new ultrasound was performed that revealed “supraclavicular adenopathy”. After that, she was referred to Secondary Healthcare (Central Hospital), where a lymph node biopsy was performed, with histological results of Kikuchi-Fujimoto disease. The patient maintains a follow-up in a hemato-oncology consultation, with painless adenopathies that, according to her, get worse with anxiety symptoms. Currently, the patient is being treated symptomatically, with stabilization of adenopathies and anxious manifestations. These patients need long-term follow-up due to the possibility of disease recurrence or the development of autoimmune processes. Although it is a diagnosis of exclusion, this disease must always be considered, since it can be mistaken with other serious pathologies that require aggressive treatments. Regarding the relationship between anxiety disorder and the worsening of adenopathies, although no conclusive evidence was found in the literature, there are some studies that have established a connection between inflammation and the deterioration of certain depressive symptoms.

## Introduction

This article was previously presented as a meeting abstract at the 2022 Update em Medicina on May 4, 2022. There are multiple cases of adenopathies that seek primary healthcare services. The differential diagnosis includes a wide spectrum of pathologies, in which the therapeutic approach varies from simple annual surveillance to aggressive therapies. Due to the significant variability in treatments, it is crucial to make a timely diagnosis, considering that this condition presents similar characteristics to other diseases such as infectious pathologies (e.g., tuberculous lymphadenitis), autoimmune disorders (e.g., systemic lupus erythematosus), or lymphoproliferative disorders (e.g., lymphoma) [1].

Kikuchi-Fujimoto disease, also known as necrotizing lymphadenitis, is a rare, benign, self-limiting entity characterized, predominantly, by a cervical lymphadenopathy that may be associated with constitutional symptoms [1]. Herein, we describe a case of a 24-year-old female patient, with no significant medical history, presenting with clinical features of right cervical lymphadenopathy, in which the histopathological examination of the excised lymph node biopsy sample allowed for the diagnosis of Kikuchi-Fujimoto disease.

## Case presentation

A 24-year-old female patient, with no significant medical history, with a family history of lymphoma in her maternal uncle and cousin, as well as cutaneous neoplasia in her maternal grandmother, resorted to the open consultation of the Family Health Unit in September 2021, due to right cervical swelling. The swelling was painful and had been progressing for two weeks, which she associated with a previous soft tissue traumatic injury. She denied any recent history of infections (including cat scratches) or constitutional symptoms such as fever or weight loss. She also denied taking any new medication during this period. Upon examination, she appeared afebrile, with a firm, tender, submandibular lateral cervical mass, measuring approximately 2 cm in its greatest diameter.

**Figure 1:**
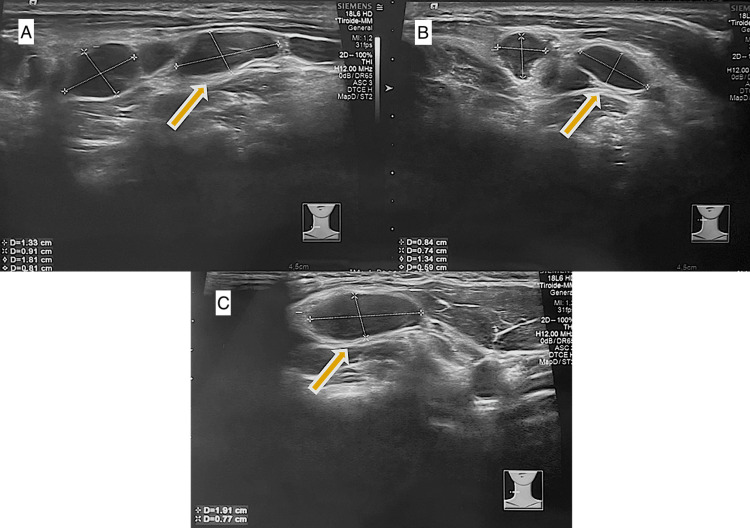
First ultrasound performed in Primary Healthcare Center First ultrasound performed in Primary Healthcare Center, showing various hypoechoic ganglion formations (A, B, C). Yellow arrow shows the largest ganglion formation measuring 19x7 mm.

A few initial investigations were ordered, including a complete blood count, protein profile, and serological tests to investigate Epstein-Barr virus (EBV) and *Toxoplasma gondii*, as well as an ultrasound of the soft tissues in the cervical region. Analytically, there was a slight increase in C-reactive protein (2.19 mg/dL), eosinophilia (6.3 x10^9^/L), and mild thrombocytopenia (124 x10^9^/L), without other relevant alterations. The serological tests were negative (Table 1), highlighting only the presence of past EBV infection. The ultrasound revealed “hypoechoic ganglion formations in the right laterocervical chains, from the retroauricular region to the lower region of the neck, the largest measuring 19x7 mm” (Figures 1A-1C).

**Table 1:** Analytical study conducted prior to diagnosis in Primary Healthcare Center AB: antibody; EBV: Epstein-Barr virus; VCA: viral capsid antigen; IgM: immunoglobulin M; IgG: immunoglobulin G.

Analytical Parameter	Result	Reference Value
Complete blood count with leukogram		
Hemoglobin	12.2 g/dL	12.0-16.0 g/dL
Erythrocytes	4.23 x10^12^/L	4.0-5.2 x10^12^/L
Platelets	124 x10^9^/L	150-400 x10^9^/L
Leukocytes	5.7 x10^9^/L	3.90-7.70 x10^9^/L
Eosinophils	6.3 x10^9^/L	0.02-0.50 x10^9^/L
Biochemistry		
C-reactive protein	2.19 mg/dL	<0.50 mg/dL
Serologies and virologies		
EBV-VCA AB - IgM	Negative	
EBV-VCA AB - IgG	Positive	
*Toxoplasma gondii* AB - IgM	Negative	
Immunology		
Hemoglobin electrophoresis	Normal	

**Figure 2:**
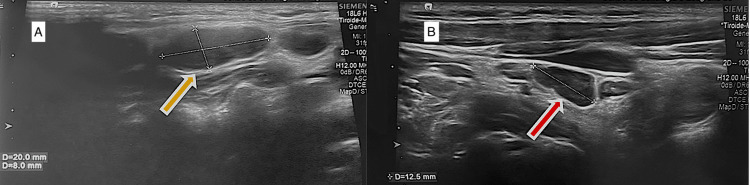
Second ultrasound performed in Primary Healthcare Center Second ultrasound performed in Primary Healthcare Center, revealing the largest hypoechoic ganglion formation (A) and supraclavicular adenopathy (B). Yellow arrow shows the largest ganglion formation measuring 20x8 mm. Red arrow shows the supraclavicular adenopathy measuring 12.5 mm.

On a follow-up after one month, the patient reported persistent symptoms with an increase in the number of lymphadenopathies, leading to a new ultrasound of the soft tissues. This new imaging exam revealed “hypoechoic ganglion formations along the right laterocervical chains, with the largest ganglion not showing significant changes in dimensions, measuring approximately 20x8 mm in its major diameter. The presence of increased lymph nodes, especially in the lower right laterocervical chains and supraclavicular region, is suspected" (Figures 2A, 2B).

Due to the persistent symptomatic presentation, the patient’s age, and the presence of supraclavicular lymphadenopathy, a referral to secondary healthcare services was made, to clarify the etiology of the clinical situation. The patient was initially evaluated by General Surgery Department in October 2021, and an excisional lymph node biopsy of the largest lymph node was performed. The histological results showed "lymph node fragments, showing traces of reactive follicles and germinal centers (CD20+, bcl2-, CD10+), with areas of necrosis, with abundant karyorrhexis phenomena, large histiocytes with eosinophilic cytoplasm, nuclei sometimes in a crescent shape, and aggregates of foamy histiocytes. A population of generally small lymphocytes with some polymorphism is associated. Immunohistochemical study shows the expression of lysozyme, CD68, myeloperoxidase (±), weak CD4, and CD123. The lymphocytes are predominantly T cells, CD3+, CD5+, and CD4+, with abundant CD8", compatible with "Kikuchi-Fujimoto disease with necrotizing and xanthomatous patterns". The patient was discharged by General Surgery Department and referred to the Hemato-oncology Department for further follow-up, where a complete analytical study was subsequently conducted (Table 2).

**Table 2:** Analytical study conducted after diagnosis in Secondary Healthcare Center, to exclude concomitant pathologies AB: antibody; IgM: immunoglobulin M; IgG: immunoglobulin G; ESR: erythrocyte sedimentation rate; HHV-8: human herpesvirus type 8; HHV-6: human herpesvirus type 6; EBV-EBNA: Epstein-Barr virus nuclear antigen; EBV: Epstein-Barr virus; VCA: viral capsid antigen; HBsAg: hepatitis B surface antigen; HBsAc: hepatitis B surface antibody; Anti-HCV: hepatitis C virus antibodies; HIV: human immunodeficiency virus; Anti-ds-DNA: antibodies against double-stranded DNA; ANA: anti-nuclear antibodies; Anti-SSA60: Sjögren's syndrome A antigen antibodies; Anti-RNP: ribonucleoprotein antibodies; Anti-Scl70: topoisomerase I enzyme antibodies; Anti-C-ANCA: antibodies against cytoplasmic antigens in neutrophils; Anti-P-ANCA: antibodies against perinuclear antigens in neutrophils; Anti-MPO: antibodies against myeloperoxidase; Anti-PR3: antibodies against proteinase 3; AMA: antimitochondrial antibodies; SMA: smooth muscle antibodies; Anti-LKM: antibodies against liver-kidney microsomes; Anti-CCP: antibodies against cyclic citrullinated peptide; Anti-TPO: antibodies against thyroid peroxidase; Anti-TG: antibodies against thyroglobulin; HLA-B*27: human leukocyte antigen-B*27 allele.

Analytical Parameter	Result	Reference Value
Complete blood count with leukogram		
Hemoglobin	12.7 g/dL	12.0-16.0 g/dL
Erythrocytes	4.49 x10^12^/L	4.0-5.2 x10^12^/L
Platelets	136 x10^9^/L	150-400 x10^9^/L
Leukocytes	6.6 x10^9^/L	3.90-7.70 x10^9^/L
Erythrocyte sedimentation rate		
Result ESR	14 mm/h	1-20 mm/h
Hemostasis		
Prothrombin time	10.8 sec	9.4-12.5 sec
Activated partial thromboplastin time	26.2 sec	23.4-35.4 sec
Biochemistry		
Adenosine deaminase	12.9 U/L	4.8-23.1 U/L
Angiotensin-converting enzyme	35 U/L	8-52 U/L
Creatinine	0.64 mg/dL	0.55-1.02 mg/dL
Total proteins	7.1 g/dL	6.6-8.3 g/dL
Albumin	3.9 g/dL	3.5-5.2 g/dL
Calcium	9.0 mg/dL	8.8-10.6 mg/dL
Lactate dehydrogenase	144 U/L	<247 U/L
Aspartate aminotransferase	16 U/L	<31 U/L
Alanine aminotransferase	9 U/L	<34 U/L
Gamma-glutamyl transferase	35 U/L	<38 U/L
Alkaline phosphatase	22 U/L	30-120 U/L
C-reactive protein	0.34 mg/dL	<0.50 mg/dL
Serologies and virologies		
HHV-8 AB - IgG	Negative	
Parvovirus B19 - DNA	Not detected	
HHV-6 - DNA	Not detected	
Cytomegalovirus AB - IgM	Negative	
Cytomegalovirus AB - IgG	Positive	
EBV-VCA AB - IgG	Positive	
EBV-VCA AB - IgG	Negative	
EBV-EBNA	Positive	
Syphilis screening - IgG/IgM	Negative	
*Toxoplasma gondii* AB - IgG	Negative	
*Toxoplasma gondii* AB - IgM	Negative	
HBsAg	Non-reactive	
HBsAc (total)	Non-reactive - immune	
Anti-HCV - IgM/IgG	Non-reactive	
HIV 1/2 (antigen and antibody)	Non-reactive	
Immunology		
Protein electrophoresis	Normal	
Immunofixation	Absence of monoclonal component	
Anti-streptolysin O	63 UI/mL	<200 UI/mL
Beta-2 microglobulin	1.50 mg/dL	0.97-2.64 mg/dL
Rheumatoid factor	<10 IU/mL	<20 IU/mL
Autoimmunity		
Anti-nuclear and anti-cytoplasmic	Negative	
Anti-ds-DNA	Negative	
Anti-SSA60	Negative	
Anti-RNP	Negative	
Anti-Scl70	Negative	
Anti-C-ANCA	Negative	
Anti-P-ANCA	Negative	
Anti-MPO	Negative	
Anti-PR3	Negative	
AMA	Negative	
SMA	Negative	
Anti-LKM	Negative	
Anti-hepatocyte cytosol	Negative	
Anti-cardiolipin - IgG/IgM	Negative	
Anti-CCP - IgG	Negative	
Anti-TPO	1.4 UI/mL	<5.6 UI/mL
Anti-TG	4.4 UI/mL	<4.0 UI/mL
Molecular biology		
HLA-B*27 allele	Not detected	

Currently, the patient still presents with painless lymphadenopathies, which, according to her, increase in size due to underlying generalized anxiety disturbance. She is on symptomatic treatment with cyclobenzaprine 10 mg once daily and paracetamol 1,000 mg as needed, along with sertraline 100 mg, started by the Family Physician, resulting in stabilization of lymphadenopathies and anxiety symptoms. The patient intends to go for psychotherapy and, till today, has not experienced recurrences or the development of other concomitant diseases.

## Discussion

The Family Physician is often the first contact for patients seeking healthcare services. Continuous knowledge updating is increasingly necessary as the pathologies encountered in primary care may be less common, requiring effective clinical reasoning. This particular disease is a rare, benign, self-limiting condition with an acute or subacute course that primarily affects young patients under 40 years of age, predominantly females, as observed in the described case [1,2]. Despite all the studies conducted to date, the etiology and pathogenesis of the disease remain unknown, and many authors are divided between two theories: the autoimmune theory and the infectious theory. The autoimmune theory suggests that the disease arises as an exaggerated immune system reaction in genetically susceptible individuals. The second theory implicates numerous infectious agents as possible etiological factors, such as cytomegalovirus (CMV), human herpesvirus (6, 7, and 8), Epstein-Barr virus, hepatitis B virus, herpes simplex virus, varicella-zoster virus, parvovirus B19, or rubella virus. Other agents such as *Toxoplasma gondii*, *Brucella*, or *Bartonella henselae* have also been suggested as possible etiological agents [2,3]. In this patient, serological studies were all negative, and she had received immunization against hepatitis B, CMV, and EBV (through natural or vaccination-induced immunity).

Most often, the initial manifestation is posterior cervical lymphadenopathy (60% to 90%), frequently accompanied by axillary and/or supraclavicular region involvement, with ganglion dimensions smaller than 3 cm in diameter, unilateral and painless. Sometimes, the appearance of lymphadenopathy in other locations must be considered and, in rare cases, they may become generalized (1% to 22% of cases). Constitutional symptoms, such as fever, are present in 35% to 77% of cases [2]. As previously described, the patient did not have constitutional symptoms, describing only unilateral but painful cervical lymphadenopathy with dimensions smaller than 3 cm, associated with supraclavicular lymphadenopathy, also characteristic of this disease.

Other symptoms commonly occur with this disease, such as asthenia, arthralgia, weight loss, hepatomegaly, myalgia, splenomegaly, nausea, or vomiting, which makes the differential diagnosis even more difficult due to their nonspecific nature [1,2]. However, in this particular case, no additional symptoms were described.

Few laboratory abnormalities can be observed such as anemia, leukopenia (in 20% to 58% of cases), leukocytosis (in 2% to 5% of cases), thrombocytopenia, increased inflammatory parameters such as C-reactive protein (CRP) and erythrocyte sedimentation rate, elevated lactate dehydrogenase, and the presence of autoimmune antibodies (such as antinuclear antibodies (ANA), antibody against the nuclear fraction of ribonucleoproteins (anti-RNP), anti-DNA, and lupus anticoagulant) [1,2]. Only three of these criteria were initially met in this patient: increased CRP, mild eosinophilia in the presence of leukocytosis, and mild thrombocytopenia, as previously presented in Table 1. A comprehensive analytical study was performed in the Secondary Healthcare Center, with negative results for all evaluated parameters.

The definitive diagnosis of this disease relies on histopathological examination, and excisional lymph node biopsy is essential [1,2], as seen in this case. This disease is often a diagnosis of exclusion, but it should always be considered a valid possibility, as it can be mistaken for other serious pathological conditions that require more aggressive treatments [1,2]. Lymphoma is a differential diagnosis that should be considered, especially in patients with a family history. In its early stages, lymphoma shares significant histopathological similarities to Kikuchi-Fujimoto disease and is misdiagnosed in 30% of cases [2,3]. Although it is a mostly benign condition, it justifies the follow-up of this patient in the Hemato-oncology Department, not only due to the family history of lymphoproliferative disease, but also due to the inherent challenges in the differential diagnosis.

The treatment of this disease is purely symptomatic (with an indication of anti-inflammatories, rest, or analgesics), and the condition resolves within a few months, with low recurrence (3% to 4%) and low associated mortality [2]. In severe cases or with persistent symptoms, a short course of low-dose corticosteroids may be indicated (e.g., deflazacort 1.5 mg/kg/day), although there is no evidence of its efficacy in the disease's progression [1].

These patients require long-term follow-up, often under the care of the Family Physician, due to the possibility of disease recurrence or the development of other autoimmune processes, such as systemic lupus erythematosus (SLE), antiphospholipid syndrome, polymyositis, thyroiditis, scleroderma, or interstitial lung disease. Due to the similarities with SLE in terms of clinical presentation, laboratory findings, and histopathological features, some authors consider Kikuchi-Fujimoto disease as a lupus-like syndrome. It has also been suggested that it may be a manifestation of SLE or may progress to it, which is the reason regular monitoring and long-term follow-up are of utmost importance [1,2].

Our research did not find any conclusive evidence linking anxiety states and the worsening of lymphadenopathies. However, some studies have established a connection between inflammation and the deterioration of certain depressive symptoms. These studies presented three main findings: elevated levels of inflammatory markers appear in patients with major depression; inflammation increases the risk of depression; and inflammatory agents may induce depressive symptoms, which can be treated with antidepressants [4]. The fact that the patient stabilized clinically with selective serotonin reuptake inhibitors can support this theory.

## Conclusions

Like this disease, there are several pathologies that require long-term follow-up, due to the possibility of recurrence or development of other related disorders, which often involves the primary healthcare resources. The Family Physician is the first point of contact for patients seeking healthcare services, and the constant updating of knowledge is increasingly necessary. This case report discusses a rare condition managed in primary healthcare. As we described, it is very important for physicians to be aware of patients' symptoms and try to fit them into these pathologies that may not be easy to diagnose, knowing that sometimes the absence of the most common symptoms could complicate the diagnosis.

## References

[1] Antunes I, Botella A, Marques F (2011). Histiocytic necrotizing lymphadenitis (Kikuchi-Fujimoto disease): a diagnostic challenge. (Article in Portuguese). Acta Med Port.

[2] Perry AM, Choi SM (2018). Kikuchi-Fujimoto disease: a review. Arch Pathol Lab Med.

[3] Ahmed Z, Quadir H, Hakobyan K, Gaddam M, Kannan A, Ojinnaka U, Mostafa JA (2021). Kikuchi-Fujimoto disease: a rare cause of cervical lymphadenopathy. Cureus.

[4] Ozen ME, Orum MH, Kalenderoglu A, Atmaca M (2019). Improvement of an atypical Kikuchi-Fujimoto disease (KFD) with antidepressant treatment: the first psychiatric approach to a KFD case. Psychiatr Clin Psychopharmacol.

